# Prognostic value of baseline clinical and HRCT findings in 101 patients with severe COVID-19 in Wuhan, China

**DOI:** 10.1038/s41598-020-74497-9

**Published:** 2020-10-16

**Authors:** Yukun Cao, Xiaoyu Han, Jin Gu, Yumin Li, Jia Liu, Osamah Alwalid, Yue Cui, Xin Zhang, Chuansheng Zheng, Yanqing Fan, Hanping Wu, Heshui Shi

**Affiliations:** 1 Department of Radiology, Union Hospital, Tongji Medical College, Huazhong University of Science and Technology, Wuhan, 430022 China; 2 Hubei Province Key Laboratory of Molecular Imaging, Wuhan, 430022 China; 3 Department of Radiology, Wuhan Jinyintan Hospital, No.1 Yintan Road, Dongxihu District, Wuhan, 430022 China; 4 Department of Radiology, Michigan Medicine, University of Michigan, Ann Arbor, MI USA

**Keywords:** Microbiology, Risk factors

## Abstract

The aim of this study was to assess the prognostic value of baseline clinical and high resolution CT (HRCT) findings in patients with severe COVID-19. In this retrospective, two-center study, we included two groups of inpatients with severe COVID-19 who had been discharged or died in Jin Yin-tan hospital and Wuhan union hospital between January 5, 2020, and February 22, 2020. Cases were confirmed by real-time polymerase chain reaction. Demographic, clinical, and laboratory data, and HRCT imaging were collected and compared between discharged and deceased patients. Univariable and multivariable logistic regression models were used to assess predictors of mortality risk in these patients. 101 patients were included in this study, of whom 66 were discharged and 35 died in the hospital. The mean age was 56.6 ± 15.1 years and 67 (66.3%) were men. Of the 101 patients, hypertension (38, 37.6%), cardiovascular disease (21,20.8%), diabetes (18,17.8%), and chronic pulmonary disease (16,15.8%) were the most common coexisting conditions. The multivariable regression analysis showed older age (OR: 1.142, 95% CI 1.059–1.231, p < 0.001), acute respiratory distress syndrome (ARDS) (OR: 10.142, 95% CI 1.611–63.853, p = 0.014), reduced lymphocyte count (OR: 0.004, 95% CI 0.001–0.306, p = 0.013), and elevated HRCT score (OR: 1.276, 95% CI 1.002–1.625, p = 0.049) to be independent predictors of mortality risk on admission in severe COVID-19 patients. These findings may have important clinical implications for decision-making based on risk stratification of severe COVID-19 patients.

## Introduction

In December 2019, a cluster of cases of pneumonia of unknown etiology, now known as coronavirus disease 2019 (COVID-19), and the coronavirus was called severe acute respiratory syndrome coronavirus 2 (SARS-CoV-2), were reported in Wuhan, Hubei province, China^1–3^. Epidemiological studies have reported that most initial patients worked at or lived around a local seafood market in Wuhan and had human to human transmission^3,4^. As of 13 September, 2020, a total of 28,584,158 COVID-19 cases have been confirmed globally, including 916,955 deaths across 200 countries, suggesting an enormous threat to the global public health^5^.

Similar to human severe acute respiratory syndrome (SARS) and middle east respiratory syndrome (MERS), SARS-CoV-2 mainly causes lower respiratory tract infections^6,7^. Previous studies have demonstrated the epidemiological, clinical characteristics and clinical outcome in patients with COVID-19, which range from mild to critically ill cases^2,3,8–10^. Yang et al.^10^ reported critically ill patients with COVID-19 having a considerable mortality, which puts tremendous pressure on hospital critical care resources. Some studies also revealed^11,12^ risk factors associated with death of adult inpatients with COVID-19, including older age, high SOFA score, coagulation dysfunction, etc. However, specific information about mortality risk factors in critically ill patients remains unclear. In addition, the MuLBSTA score^13^ indicated that the multi-lobular infiltrate assessed by chest radiography or CT imaging is the most important mortality risk factors in viral pneumonia patients. And Chen et al.^3^ reported the characteristics of deceased COVID-19 patients were in line with the score. Currently, High resolution computed tomography (HRCT) has been considered an important imaging modality in assisting the diagnosis and management of patients with COVID-19^14^. A large sample study showed that HRCT has a high sensitivity for diagnosis of COVID-19 in epidemic area^15^. However, the prognostic value of radiological findings in severe patients with COVID-19 was not reported, and previous studies^10,12^ mainly focused on chest radiography rather than the more practical HRCT imaging.

In this study, we aimed to evaluate the prognostic value of baseline clinical and HRCT findings in severe patients with COVID-19.

## Results

### Demographics and baseline characteristics in severe COVID-19 patients

Between January 5, 2020, to Feb 22, 2020, 101 patients (87 from Jin Yin-tan hospital, 14 from Wuhan Union hospital) with severe COVID-19 who underwent chest CT scans on admission were included in this study. According to the hospital data, of 87 patients from Jin Yin-tan hospital, 16 have been described by Wu et al^11^ and Zhou et al^12^.

Of all patients with severe COVID-19, 66 patients (65.3%) have recovered from severe pneumonia and were discharged from hospital, 35 cases (34.7%) died despite supportive treatment. As Table 1 showed, the mean age was 56.6 ± 15.1 years (ranged from 23 to 82 years old). 67 (66.3%) were male, 15 (14.9%) had direct exposure to the Huanan seafood market, 8 cases (7.9%) were familial clusters. The most common symptom at onset were fever (96, 95.0%) and cough (79, 78.2%). Of the 101 patients, the most common coexisting conditions was hypertension (38, 37.6%), followed by cardiovascular disease, diabetes and chronic pulmonary disease. In addition, 14 (13.9%) patients were complicated with bacterial infection.

**Table 1:** Demographics and baseline characteristics of patients with severe COVID-19.

	All patients	Survivors	Non-survivors	*P* value
	(n = 101)	(n = 66)	(n = 35)	
Age, years	56.6 ± 15.1	51.1 ± 14.2	66.8 ± 10.9	< 0.001*
**Sex**				0.430
Female	34 (33.7)	24 (36.4)	10 (28.6)	
Male	67 (66.3)	42 (63.6)	25 (71.4)	
Huanan seafood market exposure	15 (14.9)	12 (25.0)	3 (11.5)	0.217
Maximum temperature (°C)	38.7 ± 0.8	38.6 ± 0.7	38.7 ± 1.0	0.799
Heart rate (bmp)	91.7 ± 13.0	91.2 ± 12.4	92.5 ± 14.0	0.639
Respiratory rate	23.2 ± 4.9	22.4 ± 4.0	24.6 ± 5.7	0.033*
SBP (mmHg)	131.0 ± 20.0	131.7 ± 18.4	129.9 ± 23.3	0.677
DBP (mmHg)	80.1 ± 12.1	81.0 ± 11.0	78.5 ± 13.3	0.326
**Signs and symptoms**				
Fever	96 (95.0)	62 (93.9)	34 (97.1)	0.656
Fatigue	49 (48.5)	29 (43.9)	20 (57.1)	0.206
Cough	79 (78.2)	53 (80.3)	26 (74.3)	0.486
Expectoration	48 (47.5)	31 (47.0)	17 (48.6)	0.878
Dyspnea	17 (16.8)	8 (12.1)	9 (25.7)	0.082
Myalgia	13 (12.9)	8 (12.1)	5 (14.3)	0.757
Abdominal pain	2 (2.0)	0 (0)	2 (5.7)	0.118
Diarrhea	9 (8.9)	6 (9.1)	3 (8.6)	1.000
Dizziness	11 (10.9)	9 (13.6)	2 (5.7)	0.321
Nausea	9 (8.9)	4 (6.2)	5 (14.3)	0.271
Vomiting	7 (6.9)	4 (6.2)	3 (8.6)	0.693
**Comorbidities**				
Cardiovascular disease	21 (20.8)	10 (15.2)	11 (31.4)	0.055
Diabetes	18 (17.8)	6 (9.1)	12 (34.3)	0.002*
Hypertension	38 (37.6)	20 (30.3)	18 (51.4)	0.037*
Chronic pulmonary disease	16 (15.8)	9 (25.7)	7 (10.6)	0.048*
Chronic liver disease	5 (5.0)	2 (3.0)	3 (8.6)	0.338
Malignancy	5 (5.0)	3 (4.5)	2 (5.7)	0.797
Bacterial infection	14 (13.9)	8 (22.9)	6 (9.1)	0.057
Acute respiratory distress syndrome	44 (43.6)	16 (24.2)	28 (80)	< 0.001*
**Time interval from onset of symptom (days)**				
Hospital admission	11.2 ± 5.5	11.2 ± 5.5	11.3 ± 5.5	0.907
Acute respiratory distress syndrome	15.0 ± 6.6	11.9 ± 5.8	16.7 ± 6.4	0.019*
ICU admission	15.4 ± 5.4	11.9 ± 5.5	17.7 ± 5.1	< 0.001*
hospital day (days)	15.6 ± 8.1	17.1 ± 8.2	12.7 ± 7	0.008*
Duration of disease (days)	26.6 ± 8.5	28.3 ± 8.8	23.4 ± 7.1	0.006*
**Treatment**				
Oxygen therapy	101 (100)	66 (100)	35 (100)	–
Antiviral agents	88 (87.1)	64 (97.0)	24 (68.6)	< 0.001*
Antibacterial agents	93 (92.1)	60 (90.9)	33 (94.3)	0.550
Glucocorticoids	49 (50)	28 (42.4)	21 (65.6)	0.031*
Immunoglobulin	45 (86.5)	10 (58.8)	35 (100)	< 0.001*
Tracheal intubation	22 (21.8)	1 (1.5)	21 (60.0)	< 0.001*
ECMO	4 (4.1)	0 (0)	4 (12.5)	0.004*

Data are mean (SD) or n (%). p values comparing survivors and non-survivors are from χ^2^, Fisher’s exact test and independent-samples T test.

*SBP* systolic blood pressure, *DBP* diastolic blood pressure, *ICU* intensive care unit, *ECMO* extracorporeal membrane oxygenation. *p < 0.05.

The mean duration from onset of symptoms to hospital admission, ARDS and ICU admission were 11.2 ± 5.5 days, 15.0 ± 6.6 days, and 15.4 ± 5.4 days, respectively. The mean hospital day and duration of disease were 15.6 ± 8.1 days and 26.6 ± 8.5 days, respectively. The mean duration from onset of symptoms to ICU admission was significantly longer in deceased patients than discharged patients (*p* < 0.05). The hospital day and duration of disease were significantly longer in discharged patients than deceased groups (*p* < 0.05) (Table 1).

Compared with discharged patients, deceased patients were significantly older and were more likely to have ARDS and comorbidities, including cardiovascular disease, diabetes, hypertension, and chronic pulmonary disease (*p* < 0.05) (Table 1).

All patients were treated oxygen therapy (100%). 93 (92.1%) patients received antibacterial treatment, 88 (87.1%) patients received antiviral treatment, 49 (50%) received glucocorticosteroids, 45 (86.5%) received immunoglobulin, 22 (21.8%) received tracheal intubation, and 4 (4.1%) patients received extracorporeal membrane oxygenation (ECMO). Compared with discharged patients, deceased patients were more likely to receive glucocorticosteroids, immunoglobulin, tracheal intubation and ECMO (Table 1).

**Figure 1:**
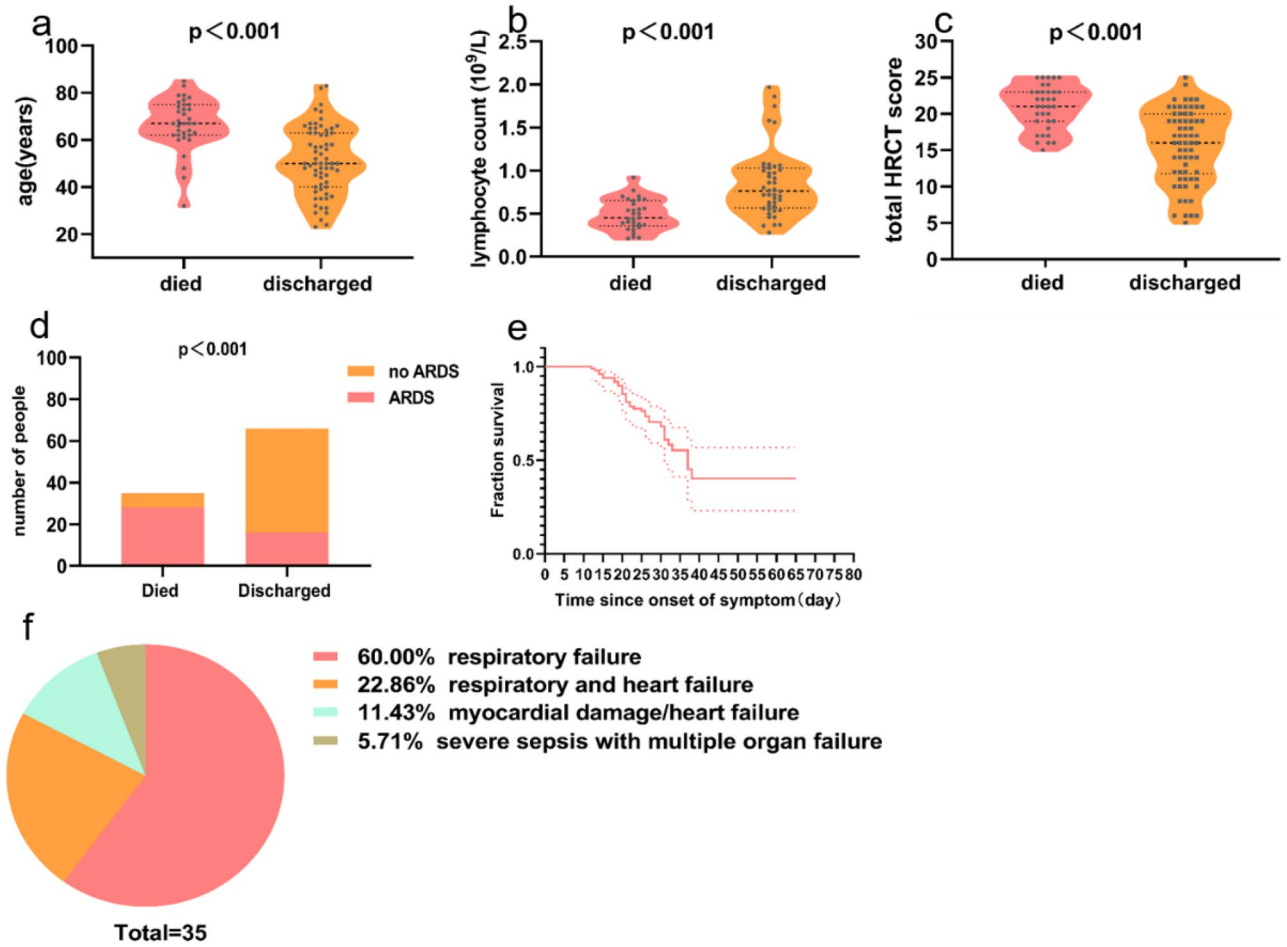
Comparison of age (**a**), lymphocyte count (**b**), total HRCT score (**c**), and ARDS proportion (**d**) between the died and discharged patients with severe COVID-19. (**e**) Survival of patients with severe COVID-19, dashed lines represent 95% CI. (**f**) Summary of the cause of death of 35 died patients with severe COVID-19.

The mean survival time in the death group from disease onset to death were 22.6 ± 7.2 days. Among the 35 severe deceased cases, 21 patients (60%) died of respiratory failure, 4 patients (11.4%) with myocardial damage died of circulatory failure, 8 patients (22.9%) died of respiratory and circulatory failure, and 2 (5.7%) with severe sepsis died of multiple organ failure (Fig. 1).

### Baseline laboratory findings in severe COVID-19 patients

The laboratory findings showed the levels of hyper-sensitive C-reactive protein, Serum amyloid A protein, and erythrocyte sedimentation rate were markedly increased in almost all patients (Table 2). The lymphocyte count, hemoglobin, albumin level on admission and oxygen saturation on room air were significantly lower in deceased patients than discharged patients (*p* < 0.05). Lactate dehydrogenase (LDH), creatinine, D-dimer and hypersensitive troponin I level on admission were higher in deceased patients than discharged patients (*p* < 0.05) (Table 2).

**Table 2:** Baseline laboratory findings of patients with severe COVID-19.

	All patients	Survivors	Non-survivors	Normal range	P value
	(n = 101)	(n = 66)	(n = 35)		
Leukocyte count (10^9^/L)	7.6 ± 3.9	7.6 ± 3.9	7.5 ± 4.0	4.0 ~ 10.0	0.971
Neutrophil Count (10^9^/L)	7.2 ± 3.7	7.3 ± 3.8	7.0 ± 3.6	1.8 ~ 6.3	0.852
Lymphocyte count (10^9^/L)	0.70 ± 0.37	0.85 ± 0.39	0.49 ± 0.18	1.1 ~ 3.2	< 0.001*
Platelet count (10^9^/L)	164.5 ± 52.1	169.4 ± 53.2	156.3 ± 50.2	125.0 ~ 350.0	0.333
Hemoglobin (g/L)	120.4 ± 18.5	123.7 ± 16.6	114.8 ± 20.5	130.0 ~ 175.0	0.046*
Hyper-sensitive C-reactive protein (mg/L)	67.3 (24.0, 133.9)	54.3(16.0, 129.6)	90.4 (45.1, 141.0)	< 25.0	0.057
Serum amyloid A protein (mg/L)	174.3 ± 86.6	165.0 ± 94.5	193.9 ± 64.0	< 10.0	0.170
Erythrocyte sedimentation rate (mm/h)	53.0 ± 25.1	52.3 ± 23.4	54.3 ± 28.1	0 ~ 15.0	0.738
Interleukin-6 (pg/ml)	11.6 ± 9.4	10.9 ± 9.9	12.9 ± 8.5	0.1 ~ 2.9	0.407
ALT (U/L)	54.3 ± 33.3	57.4 ± 33.3	49.2 ± 33.4	5.0 ~ 40.0	0.310
AST (U/L)	58.8 ± 36.9	56.6 ± 33.6	62.5 ± 42.0	8.0 ~ 40.0	0.501
Albumin (g/L)	31.0 ± 4.4	32.2 ± 4.0	28.3 ± 4.2	35.0 ~ 55.0	< 0.001*
Lactate dehydrogenase (U/L)	476.3 ± 243.4	402.9 ± 163.8	583.8 ± 299.9	109.0 ~ 254.0	0.001*
Glucose (mmol/L)	8.5 ± 5.0	8.3 ± 5.7	8.7 ± 3.8	3.9 ~ 6.1	0.718
Creatinine (umol/L)	80.0 (66.5, 99.8)	75.0 (65.2, 83.6)	93.0 (74.2, 125.3)	44.0 ~ 133.0	0.017*
Prothrombin time (s)	16.8 (16.2, 21.1)	16.8 (16.2, 23.1)	17.7 (16.1, 20.7)	11.0 ~ 16.0	0.691
Activated partial thromboplastin time(s)	26.1 ± 8.9	24.5 ± 8.3	28.3 ± 9.4	28.0 ~ 43.5	0.189
Thrombin time (s)	19.0 ± 7.2	18.2 ± 5.7	20.0 ± 9.7	14.0 ~ 21.0	0.463
D-dimer (mg/L)	3.09 (0.8, 7.1)	1.5 (0.6, 3.1)	7.0 (3.3, 28.0)	< 0.5	< 0.001*
Hypersensitive troponin I(pg/mL)	15.3 (3.4, 37.7)	3.6 (0.3, 18.9)	31.4 (11.0, 94.2)	< 0.6	< 0.001*
Myoglobin (ug/L)	118.3 (54.3, 189.2)	65.5 (38.7, 183.3)	141.3 (59.3, 194.8)	50.0 ~ 85.0	0.141
Oxygen saturation on room air (%)	87.7 ± 9.7	90.7 ± 8.5	84.1 ± 9.9	95.0 ~ 99.0	0.005*
PH	7.5 ± 0.1	7.5 ± 0.05	7.4 ± 0.1	7.35 ~ 7.45	0.168
PO_2_ (mmHg)	70.6 ± 18.2	71.6 ± 20.9	69.3 ± 14.2	80 ~ 100	0.665
PCO_2_ (mmHg)	35.7 ± 11.8	36.4 ± 12.4	34.7 ± 11.2	35 ~ 45	0.638

Data are median (IQR), mean (SD) or n (%). p values comparing Non-survivors and survivors are from χ^2^, Fisher’s exact test, independent-samples T test or Mann–Whitney U test.

*ALT* alanine transaminase, *AST* aspartate aminotransferase.

*p < 0.05.

### Baseline HRCT findings in severe COVID-19 patients

**Figure 2:**
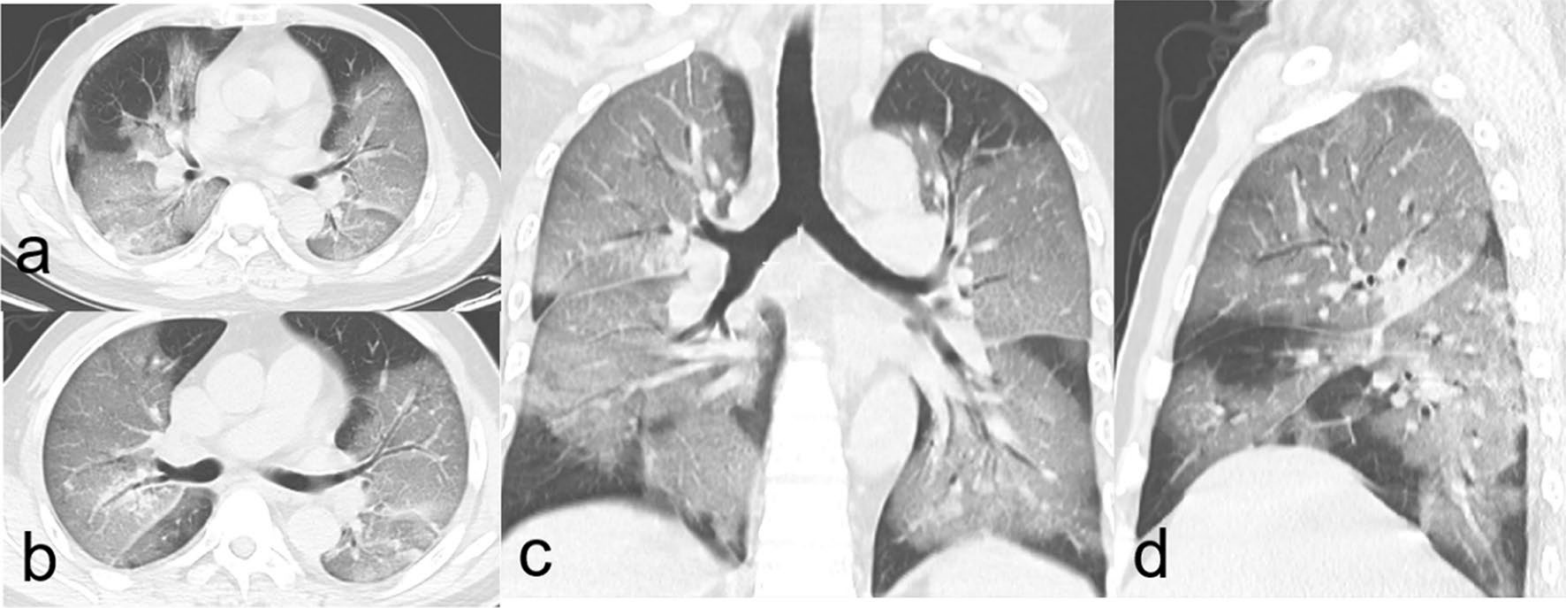
Thin-section CT scans in a man infected with SARS-CoV-2. Scan obtained on 7th day from onset of symptoms shows diffuse ground glass opacities that affected bilateral lung parenchyma. The patient died 10 days after this scan due to respiratory failure.

**Figure 3:**
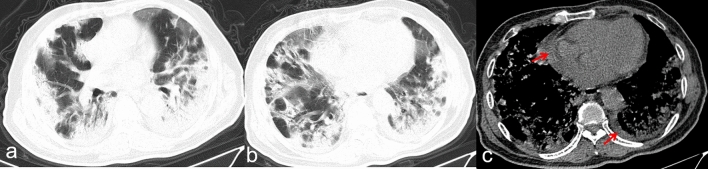
Transverse CT scans in a man infected with SARS-CoV-2 and with type 2 diabetes for 20 years. Scan obtained on 19th day from onset of symptoms shows diffuse, heterogeneous consolidation that affected bilateral lung parenchyma (**a**,**b**). The mediastinal window (**c**) shows a small pleural effusion at the right (arrow) and a small pericardial effusion (arrow). The patient died 4 days after this scan due to respiratory failure.

**Figure 4:**
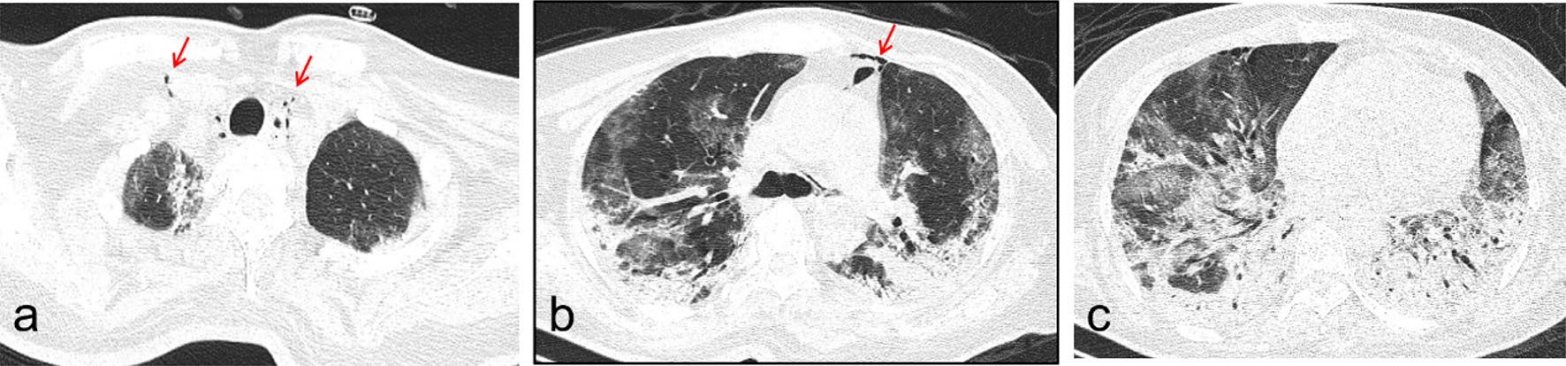
Transverse CT scans in a woman infected with SARS-CoV-2 and suffering type 2 diabetes for 10 years. Scan obtained on 16th day from onset of symptoms shows bilateral, diffuse consolidation, which predominantly involved the lower lobes. There was pneumomediastinum were also seen (**a**,**b** arrows). The patient died 7 days after this scan because of both circulatory and respiratory failure.

**Figure 5:**
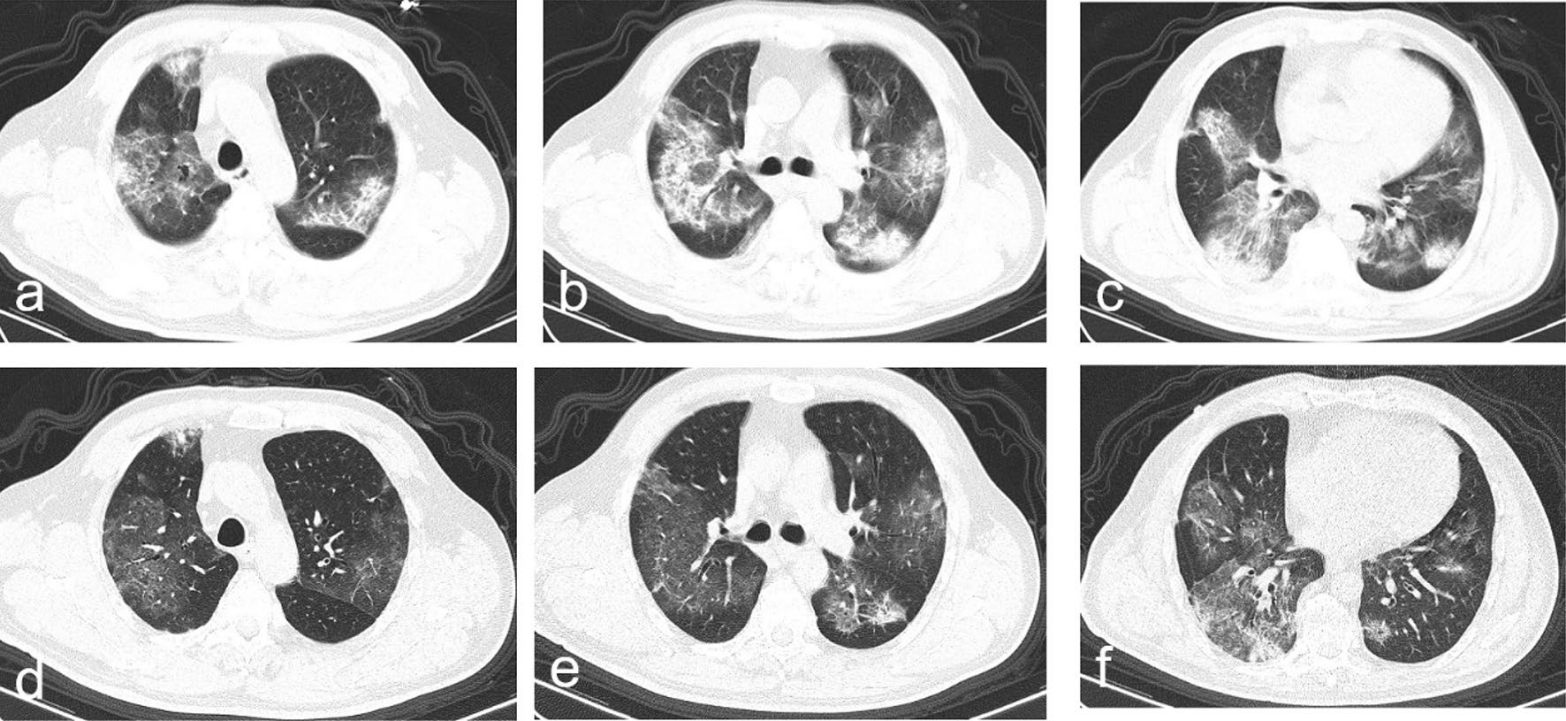
Transverse CT scans in a man with infected with SARS-CoV-2. Scan (**a**–**c**) obtained on 9th day from onset of symptoms shows bilateral, consolidation combined with ground glass opacities. Scan (**d**–**f**) obtained on 20th day shows previous consolidation being dissipated into ground-glass opacities. The patient was discharged from hospital 6 days after the final scan.

**Figure 6:**
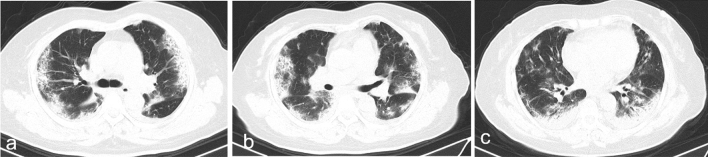
Transverse CT scans in a man infected with SARS-CoV-2. Scan obtained on 10th day from onset of symptoms shows diffuse, heterogeneous consolidation that affected bilateral, subpleural lung parenchyma. The patient was discharged from hospital 7 days the scan.

**Figure 7:**
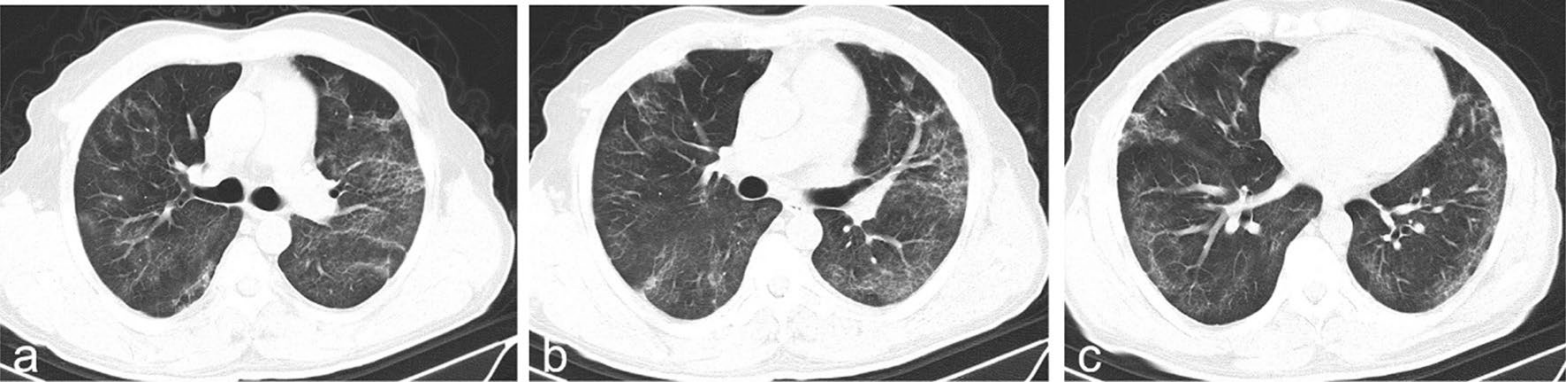
Transverse CT scans in a man with infected with SARS-CoV-2. Scan obtained on 20th day from onset of symptoms shows bilateral, light ground glass opacities with irregular linear opacities. The patient was discharged from hospital 11 days after the scan.

The median time interval from admission to baseline CT scan in all patients were 4 days (IQR 1-9), with no difference between discharged and deceased patients (Table 3). The typical chest CT findings of severe COVID-19 on admission were diffuse bilateral GGO and consolidation in peripheral areas (Figs. 2, 3, 4, 5, 6, 7). GGO (90, 89.1%) is main diffusion lesion characteristics in all patients, and consolidation proportion (8 [22.9] vs 3 [4.5], *p *= 0.005) was comparatively higher in deceased patients than discharged patients (Table 3). The mean total CT scores in all patients was 17.4 ± 5.1. Deceased patients had higher CT scores than discharged patients (20.9 ± 3.0 vs 15.6 ± 5.0, *p* < 0.001) (Fig. 1).

**Table 3:** Baseline HRCT findings of patients with severe COVID-19.

	All patients	Survivors	Non-survivors	*P* value
	(n = 101)	(n = 66)	(n = 35)	
Time from admission to CT scan, days	4 (1–9)	4 (1–9)	5 (3–9)	0.436
**HRCT score**				–
Left upper lobe	3.4 ± 1.3	3.0 ± 1.4	4.1 ± 1.0	< 0.001*
Left lower lobe	3.7 ± 1.2	3.4 ± 1.3	4.2 ± 1.0	0.001*
Right upper lobe	3.5 ± 1.5	3.1 ± 1.4	4.4 ± 1.2	< 0.001*
Right middle lobe	3.0 ± 1.3	2.6 ± 1.2	3.8 ± 1.0	< 0.001*
Right lower lobe	3.8 ± 1.1	3.5 ± 1.2	4.4 ± 0.9	< 0.001*
Total lesions score	17.4 ± 5.1	15.6 ± 5.0	20.9 ± 3.0	< 0.001*
**Lung involvement**				–
Unilateral	0	0	0	–
Bilateral	101 (100)	66 (100)	35 (100)	–
**Predominant distribution**				–
Diffusely septal/subpleural area	101 (100)	66 (100)	35 (100)	–
Diffusely central hilar area	0	0	0	–
**Main lesion component**				0.005*
GGO	90 (89.1)	63 (95.5)	27 (77.1)	–
Consolidation	11 (10.9)	3 (4.5)	8 (22.9)	–
**Other typical signs**				–
Interlobular septal thickening	72 (71.3)	40 (60.6)	32 (91.4)	0.001*
Crazy-paving sign	48 (47.5)	25 (37.9)	23 (65.7)	0.008*
Air bronchogram	75 (74.3)	40 (60.6)	35 (100)	< 0.001*
**Coexisting lesion**				–
Pleural effusion	28 (28.0)	11 (16.9)	17 (48.6)	0.001*
Emphysema	15 (14.9)	7 (10.6)	8 (22.9)	0.099
Hydropericardium	10 (9.9)	5 (7.6)	5 (14.3)	0.283
Pneumomediastinum	3 (3.0)	2 (3.0)	1 (2.9)	1.000

Data are mean (SD) or n (%). p values comparing discharged patients and died patients are from χ^2^, Fisher’s exact test, or independent-samples T test.

*GGO* ground-glass opacities.

*p < 0.05.

The other common CT findings were interlobular septal thickening (72/101, 71.3%), crazy paving (48/101, 47.5%) and air bronchograms (75/101, 74.3%). The relatively less common CT findings were pleural effusion (28/101, 28%), emphysema (15/101, 14.9%), hydropericardium (10/101, 9.9%) and pneumomediastinum (3/101, 3.0%) (Table 3).

Interlobular septal thickening, crazy-paving, air bronchogram, and pleural effusion were significantly more common in deceased patients than discharged patients (*p* < 0.05) (Table 3).

### Univariate and multivariable analysis of predictors of mortality risk

By univariate regression analysis in severe COVID-19 patients, the following baseline characteristics were predictors of mortality risk: older age, faster respiratory rate, ARDS, history of diabetes, history of hypertension; the following laboratory findings were predictors of mortality risk: reduced lymphocyte count, reduced albumin, elevated lactate dehydrogenase, elevated D-dimer, reduced SpO2 at room air; the following baseline HRCT findings were predictors of mortality risk: elevated total HRCT score, higher consolidation proportion, pleural effusion (Table 4).

**Table 4:** Univariate analysis of predictors of mortality risk in patients with severe COVID-19.

	Odds ratio	95% CI	P value
Age	1.102	1.055–1.151	< 0.001*
Sex	1.429	0.588–3.473	0.431
Huanan seafood market exposure	0.391	0.100–1.538	0.179
Maximum temperature at admission	1.071	0.638–1.796	0.796
Heart rate at admission	1.008	0.976–1.040	0.636
Respiratory rate	1.095	1.001–1.198	0.048*
SBP	0.996	0.975–1.016	0.674
DBP	0.983	0.950–1.017	0.324
Fever	2.194	0.236–20.417	0.490
Fatigue	1.701	0.744–3.891	0.208
Cough	0.709	0.268–1.871	0.487
Expectoration	1.066	0.469–2.422	0.878
Dyspnea	2.510	0.871–7.235	0.089
Myalgia	1.208	0.364–4.016	0.757
Diarrhea	0.938	0.220–4.000	0.931
Dizziness	0.384	0.078–1.884	0.238
Nausea	2.542	0.636–10.159	0.187
Vomiting	1.430	0.301–6.782	0.653
Acute respiratory distress syndrome	12.500	4.592–34.028	< 0.001*
Cardiovascular disease	2.567	0.962–6.844	0.060
Diabetes	5.217	1.752–15.542	0.003*
Hypertension	2.435	1.046–5.672	0.039*
Chronic pulmonary disease	2.918	0.981–8.679	0.054
Chronic liver disease	3.000	0.477–18.867	0.242
Malignancy	1.273	0.203–7.999	0.797
Bacterial infection	2.963	0.936–9.375	0.065
Leukocyte count	0.998	0.888–1.121	0.971
Lymphocyte count	0.003	0.000–0.064	< 0.001*
Neutrophil count	0.982	0.815–1.183	0.847
Platelet count	0.995	0.985–1.005	0.329
Hemoglobin	0.973	0.946–1.000	0.052
Hyper-sensitive C-reactive protein	1.006	0.999–1.013	0.113
Serum amyloid A protein	1.004	0.998–1.010	0.171
Erythrocyte sedimentation rate	1.003	0.985–1.022	0.734
Interleukin-6	1.023	0.969–1.079	0.415
ALT	0.992	0.977–1.007	0.307
AST	1.004	0.992–1.017	0.498
Albumin	0.790	0.694–0.899	< 0.001*
Lactate dehydrogenase	1.004	1.001–1.006	0.004*
Glucose	1.018	0.926–1.118	0.716
Creatinine	1.004	0.997–1.010	0.257
Activated partial thromboplastin time	1.051	0.974–1.134	0.200
Thrombin time	1.039	0.938–1.150	0.464
D-dimer	1.039	1.004–1.076	0.031*
Hypersensitive troponin I	1.001	0.998–1.003	0.620
Myoglobin	1.001	0.997–1.005	0.749
Oxygen saturation on room air	0.916	0.855–0.981	0.012*
PH	0.001	0.001–7.861	0.135
PO_2_	0.993	0.963–1.024	0.658
PCO_2_	0.987	0.933–1.043	0.633
Total lesions score	1.389	1.196–1.613	< 0.001*
Main lesion component (GGO/consolidation)	6.222	1.532–25.268	0.011*
Pleural effusion	4.636	1.834–11.718	0.001*
Emphysema	2.497	0.821–7.592	0.107
Hydropericardium	0.941	0.082–10.757	0.961
Pneumomediastinum	2.033	0.546–7.569	0.290

*SBP* systolic blood pressure, *DBP* diastolic blood pressure, *ALT* alanine transaminase, *AST* aspartate aminotransferase.

*p < 0.05.

The multivariable regression analysis showed older age (OR: 1.142, 95% CI 1.059–1.231, *p* < 0.001), ARDS (OR: 10.142, 95% CI 1.611–63.853, *p* = 0.014), reduced lymphocyte count (OR: 0.004, 95% CI 0.001–0.306, *p* = 0.013), and elevated HRCT score (OR: 1.276, 95% CI 1.002–1.625, *p* = 0.049) independent predictors of mortality risk on admission in severe COVID-19 patients (Table 5).

**Table 5:** Multivariable study of predictors of mortality risk in patients with severe COVID-19.

	Odds ratio	95% CI	P values
**Model 1: baseline characteristics**			
Age	1.154	1.068–1.248	< 0.001*
Respiratory rate	1.127	0.990–1.284	0.171
Dyspnea	1.257	0.218–7.259	0.798
Acute respiratory distress syndrome	14.951	3.539–63.171	< 0.001*
Cardiovascular disease	0.439	0.081–2.387	0.341
Diabetes	1.239	0.231–6.638	0.802
Hypertension	0.733	0.151–3.562	0.700
Chronic pulmonary disease	1.795	0.323–9.969	0.504
Bacterial infection	3.257	0.558–19.031	0.190
**Model 2: laboratory findings**			
Lymphocyte count	0.001	0.001–0.194	0.019*
Hemoglobin	0.914	0.800–1.044	0.183
Albumin	0.872	0.586–1.299	0.501
Lactate dehydrogenase	1.004	0.996–1.013	0.305
D-dimer	0.981	0.925–1.041	0.530
Oxygen saturation on room air	0.914	0.800–1.044	0.183
**Model 3: HRCT findings**			
Total lesions score	1.332	1.138–1.559	< 0.001*
Main lesion component (GGO/consolidation)	3.408	0.710–16.360	0.126
Pleural effusion	2.070	0.693–6.184	0.193
Emphysema	2.581	0.628–10.610	0.189
**Model 4: baseline characteristics + Laboratory findings + HRCT findings**			
Age	1.142	1.059–1.231	0.001*
Acute respiratory distress syndrome	10.142	1.611–63.853	0.014*
Lymphocyte count	0.004	0.001–0.306	0.013*
Total lesions score	1.276	1.002–1.625	0.049*

*p < 0.05.

## Discussion

In this study, we have investigated the prognostic value of baseline clinical and HRCT findings in severe patients with COVID-19. In two previously published prognostic studies^11,12^, included groups were not all severe patients and HRCT findings associated with poor clinical outcomes have not been specified. The main finding of our study was that older age, developed ARDS, lymphocytopenia, and high CT score were the independent predictors of mortality risk in severe patients with COVID-19.

In our study, the mean age of severe patients with COVID-19 was 56.6 ± 15.1 years old, and more than 60% of patients were men. As described in previous studies^2,3,9,10^, elderly male patients are more susceptible to SARS-CoV-2 infection, which were supported by our severe patient cohort. But there was no sexual distinction between deceased and discharged patients in our study, which indicated gender is not a risk factor of death. In concert with a recent study^10^, we observed that deceased group was significantly older than the discharged group in severe COVID-19. And in the multivariable regression analysis, we have demonstrated the older age was an independent predictor of mortality risk in severe COVID-19 patients. Therefore, the clinical imperatively management for severe elderly patients requires further attention. Fever is the most common clinical symptom in severe patients with COVID-19, but 5% patients had no fever at the onset of disease, which is also in line with previous studies^8,16^. If patients are asymptomatic SARS-CoV-2 carriers, early diagnosis is relatively difficult for areas with less medical specialties^1^. Absence of typical initial symptoms for few patients may delay the optimal quarantine and treatment time and lead to poor outcome. However, in our present study, no significant differences were found between the initial symptoms of deceased patients and discharged patients.

ARDS characterized by an acute, diffuse, inflammatory lung damage is one of the most common causes of respiratory failure in critically ill patients with viral pneumonia^17^. In our study, compared with discharged patients with severe COVID-19, the deceased group has significantly higher incidence of ARDS. ARDS was an independent predictor of mortality risk in severe COVID-19 patients, which is similar to critically ill patients with SARS^18^. The incidence of ARDS in the present study is consistent with previous critically ill patients with COVID-19^10^, but which is slightly higher than that previously seen in patients with SARS^18,19^ and MERS^20^. Such a discrepancy may be due to failure to timely diagnosis and treatment in the situation of sudden increase in infected patients. Of note, the incidence of ARDS of survivors in the present study is significantly higher than that recently reported in Zhou’s study^12^. The different inclusion criteria, our discharged group were all severe patients, may result in the problem. In future, the earlier recognition of ARDS and ongoing efforts to study potential mechanisms of pulmonary damage in severe COVID-19 are the keys to reduce mortality rate. In addition, hypertension, cardiovascular disease and diabetes are the most common comorbidities in severe patients, which is in keep with previous studies^9,10^. Similarly, deceased patients had more comorbidities, such as hypertension, diabetes, and chronic pulmonary disease, than those discharged. Although comorbidities were not risk factors for death event in multivariable regression analysis, clinicians still require to focus on the clinical progression in severe patients.

In terms of laboratory indexes, more than 80% of severe patients in the present study have lymphocytopenia on admission, and the lymphocyte count was significantly lower in deceased patients than discharged patients. This finding is similar to previous COVID-19, SARS and MERS patients^10,21,22^. Unlike Chen’s study showed less than 40% of non-severe patients had only mild lymphocytopenia, the present study indicated the extent of lymphocytopenia may reflect the severity of SARS-CoV-2 infection. Furthermore, our study first demonstrated lymphocytopenia is an independent predictor of mortality risk in severe patients with COVID-19. Prior studies reported that severe SARS or MERS patients were prone to have lymphocytopenia. It may due to virus particle directly invading and damaging the lymphocyte cytoplasmic component or lymphocytes apoptosis^16,22^. Unfortunately, the pathogenesis of lymphocytopenia in severe COVID-19 is still unclear by now, later basic immunology may be the key to solve the problem. Additionally, compared with discharged patients, deceased patients had lower hemoglobin and albumin level, higher LDH, Creatinine, D-dimer and hypersensitive troponin I level. These abnormal results suggest that the severity of SARS-CoV-2 infection may be related to coagulation activation, myocardial damage and kidney damage. Unlike a previous study^9^, although more than 50% severe patients have elevated ALT and AST, no significant differences of ALT and AST level were found between deceased and discharged patients with severe COVID-19. Liver damage in patients with COVID-19 is common, especially in critically ill patients. The possible reasons are as follows: (1) liver cells are possibly directly infected with virus, (2) drug hepatotoxicity in the treatment, (3) immune-mediated inflammation, such as cytokine storm^23^. Although, liver damage is not a prognostic factor in the present study, further research still should pay close attention to the causes and evolution of liver injury in COVID-19.

Of note, the present study showed that elevated baseline HRCT score is an independent prognostic factor of mortality risk in severe COVID-19 patients. CT score^24^ was a semi quantitative parameter, which could quantify the severity of pulmonary abnormalities (GGO and consolidation dominated in COVID-19). Previous studies^22,25^ have demonstrated that the score was correlated with the degree of pulmonary lesions in pathologic specimens. CT score has been applied in the quantitative assessment of the evolution of pulmonary lesions in previous SARS^26^ and COVID-19 patients^27^. To the best of our knowledge, this is the first study to assess the prognostic value of baseline CT score, which has a significant value for clinical decisions based on radiological risk stratification in severe COVID-19 patients. We have reasons to believe that CT scan after hospital admission may be an important imaging modality in assisting the diagnosis and assessment of outcome in patients with severe COVID-19. However, given the infectious risk of transporting patients with COVID-19 for other hospitalized patients and health care workers, it's necessary to select the appropriate timing for immediate CT imaging. First, in the outbreak stage of COVID-19, RT-PCR for SARS-CoV-2 viral nucleic acid was reported to have a sensitivity of less than 60%^15,28^. Based on a higher sensitivity (80–90%) for COVID-19^15,29^, HRCT can be considered as a primary tool for the current COVID-19 detection in epidemic areas. Second, With the improvement of the accuracy and rapidity of the RT-PCR testing, immediate CT imaging is mainly used for suspected positive patients^30,31^. In addition, there were no pregnant women among the study, however, according to previous studies, HRCT examination for pregnant patients may be performed in the appropriate clinical settings^32^. Given the concern of radiation exposure to the fetus, for asymptomatic pregnant patients, self-quarantine until the results of RT‐PCR (not CT) are available may be considered. For pregnant patients who are prone to severe COVID-19, CT scan after hospital admission may be an important tool in assisting reasonable risk stratification and timely clinical management.

Like SARS and MERS, the CT findings of severe COVID-19 patients were nonspecific, which included diffusely bilateral GGO and consolidation of subsegmental areas. However, the extent of consolidation in severe COVID-19 patients was less severe than SARS patients^33^. Interestingly, although GGO was mainly diffuse in all patients, the consolidation proportion was comparatively higher in died patients than discharged patients. In a recent autopsy report of a deceased COVID-19 patient, Liu et al.^34^ demonstrated that edema, inflammatory infiltrate, and exudation were common in affected lung areas. Those may explain the predominant pattern of GGO on CT images^35^. At present, the relation between the extent of consolidation and outcome is unclear, which needs further large sample autopsy and HRCT studies. In this study, 28% patients with severe COVID-19 had pleural effusion, and which was significantly more common in deceased patients than discharged patients. Due to the present study mainly included severe patients, the incidence of pleural effusion may be higher than previous studies^36–38^. Das et al. study^39^ showed that the presence of pleural effusion was a poor prognostic factor in patients with MERS. Consequently, severe patients having abnormal pleural effusion deserve the attention of clinicians. All in all, baseline CT may be an important imaging modality in assisting the diagnosis and assessment of outcome in patients with severe COVID-19.

This study has several limitations. First, the sample size was small. Second, this is a retrospective study design, a few of clinical and laboratory results were missing in some patients, which may lead to unavoidable biases in analysis. Third, some survivors still hospitalized were not included the present study, the mortality rate of severe patients was overestimated in this study.

In conclusion, older age, ARDS, lymphocytopenia and elevated CT score were strong predictors of mortality risk in severe COVID-19 patients. Patients who are prone to severe COVID-19, CT scan after hospital admission may be an important imaging modality for assisting assessment of outcome in patients with severe COVID-19. Our findings may have important implications for clinical decisions based on risk stratification in severe pneumonia patients. As our study was retrospectively designed with possible selection bias, further prospective studies are needed to confirm our findings.

## Materials and methods

### Study design and participants

A retrospective two-center study was performed in Wuhan Jin Yin-tan hospital and Wuhan Union hospital (Wuhan, Hubei province, China). From January 5, 2020, to Feb 22, 2020, we enrolled two groups of patients (deceased and discharged patients) with severe COVID-19, who underwent HRCT scan at admission. All patients of confirmed infection with SARS-CoV-2 are in accordance with World Health Organization (WHO) interim guidance^40^. Throat swab samples were collected for confirmation of SARS-CoV-2 by the real-time polymerase chain reaction assay as previously described^2,3^. The diagnostic criteria for adult severe pneumonia was according to WHO interim guidance^41^, which includes fever or suspected respiratory tract infection, plus one of followings: respiratory rate > 30 breaths/min, SpO2 < 90% on room air or severe respiratory distress. The discharge criteria for patients was according to the sixth edition of “pneumonia diagnosis and treatment plan for new coronavirus infection” in China^42^, which includes normal temperature for more than 3 days, both the respiratory symptoms and chest imaging showing significant improvement, and negative two consecutive respiratory pathogen nucleic acid tests with the interval at least 1 day. This study had ethics approval of the Ethics Commission of Wuhan Jin Yin-tan hospital and Wuhan Union hospital. All participants remained anonymous, and written informed consent was waived by ethics commission for rapid emerging infectious diseases. This study was conducted in compliance with the Declaration of Helsinki.

### Clinical data collection

We reviewed the electronic medical records, nursing records, laboratory findings, and radiology findings in all patients. All data were independently reviewed and checked by four physicians (J.L., Y.L., X.H., Y.C.). We collected age, sex, exposure history, underlying disease, maximum body temperature, vital signs, onset of symptoms, and the laboratory finding at admission (complete blood count, liver and kidney function, coagulation profile, C-reactive protein, erythrocyte sedimentation rate, blood gas analysis etc*.*) and treatment measures [oxygen therapy, antiviral agents, antibacterial agents, corticosteroids, immunoglobulin, tracheal intubation and extracorporeal membrane oxygenation (ECMO)]. In addition**,** the durations from onset of disease to hospital admission, chest HRCT scan, ARDS and ICU admission were recorded. ARDS was diagnosed according to the Berlin definition^43^.

### HRCT image acquisition

All patients underwent HRCT examination in the supine position on one of the three CT scanners: SOMATOM Definition AS+, SOMATOM Perspective, or SOMATOM Spirit (Siemens Healthineers, Germany). Scans were done from the thoracic inlet to the diaphragm, and no contrast medium was used. CT scan parameters were as described in our previous study^38^. All images were reconstructed using standard reconstruction algorithms with a slice thickness of 2 mm or 5 mm. The multiplanar reconstruction postprocessing was implemented on workstation and picture archiving and communication systems.

### HRCT image interpretation

All HRCT images were reviewed in random order by three radiologists (H.S., J.G., Y.F.) who were senior cardiothoracic radiologists. The readers independently assessed the CT features using both axial and MPR images, who were unaware of any other clinical and laboratory findings or outcome of the patients. After independent evaluation, discussion and consensus resolved the disagreements.

For each severe pneumonia patients, the predominant HRCT patterns defined by the Fleischner Society glossary^44^ were as following: ground-glass opacities (GGO), consolidation, interlobular septal thickening, crazy-paving pattern, air bronchogram, pneumonectasis, pleural effusion and pneumomediastinum. The distribution of lesion was categorized as diffuse central area and peripheral area.

To quantify the extent of pulmonary abnormalities, a HRCT score system^24^ was assigned on the basis of the area involved. And a previous study^22^ has demonstrated that this system was correlated well with the degree of pulmonary lesions in pathologic specimens. Specifically, each of the five lung lobes was visually scored from 0 to 5 as: 0, no involvement; (1) < 5% of lobe (minimal but not normal), (2) 5–25% of lobe, (3) 26–49% of lobe; (4) 50–75% of lobe; (5) > 75% of lobe. Finally, the total CT severity score was calculated by summing the individual lobar scores (range of possible scores from 0 to 25).

### Statistical analysis

The Kolmogorov–Smirnov test was used to checked the normality of all continuous data. Normally and non-normally distributed data and categorical variables are expressed as the mean (SD) and the medians (IQR) and number (%), respectively. We assessed differences between two groups of normally distributed variables using two-sample t test or Mann–Whitney U test depending on normally and non-normally distributed data for continuous variables and Fisher’s exact test for categorical variables. The association between baseline clinical or HRCT Findings and incidence of death events was assessed with univariate logistic regression model (P < 0.1 in Tables 1, 2, 3). From a clinical point of view, to ensure proper independent variable number and avoid variable overfitting of the final multivariable model, we implemented the following step: (1) first, a multivariable model (Model 1: baseline characteristics) was tested, including those baseline variables that showed an association (p < 0.1 in Table 4) with incidence of death events. (2) A second multivariable model (Model 2: laboratory findings) was tested, including those baseline laboratory findings that showed an association (p < 0.1 in Table 4) with incidence of death events. (3) A third multivariable model (Model 3: HRCT findings) was tested, including those baseline HRCT findings that showed an association (p < 0.1 in Table 4) with incidence of death events. (4) A final multivariable model (Model 4: baseline characteristics plus laboratory findings plus HRCT findings) included variables from Model 1–3 that were independently related to the incidence of death events. The minimum sample size of the study should be 10 times greater than the included independent variable number. Co-linearity of variables tested in multivariate regression model (Table 4) was evaluated using the tolerance statistic and the variance inflation factor (excessive if < 0.1 and if > 10, respectively). Statistical significance was considered a p value < 0.05 (2-tailed). Analyses were conducted using SPSS software (SPSS 21.0 for Windows, IBM, Chicago, IL, USA).

### Ethics declarations

This study was approved by both ethics committees of Union hospital of Tongji Medical College of Huazhong University of Science and Technology and Wuhan Jin-yintan hospital. The written informed consent was waived by ethics commission for rapid emerging infectious diseases.

## Data Availability

The datasets used and analyzed during the current study are available from the corresponding author on reasonable request.
